# A Rapid and Sensitive Method for Measuring N-Acetylglucosaminidase Activity in Cultured Cells

**DOI:** 10.1371/journal.pone.0068060

**Published:** 2013-06-28

**Authors:** Victor Mauri, Parisa Lotfi, Laura Segatori, Marco Sardiello

**Affiliations:** 1 Department of Molecular and Human Genetics, Baylor College of Medicine, Jan and Dan Duncan Neurological Research Institute, Texas Children’s Hospital, Houston, Texas, United States of America; 2 Departments of Chemical and Biomolecular Engineering, Biochemistry and Cell Biology, and Bioengineering, Rice University, Houston, Texas, United States of America; University Hospital S. Maria della Misericordia, Udine, Italy

## Abstract

A rapid and sensitive method to quantitatively assess N-acetylglucosaminidase (NAG) activity in cultured cells is highly desirable for both basic research and clinical studies. NAG activity is deficient in cells from patients with Mucopolysaccharidosis type IIIB (MPS IIIB) due to mutations in *NAGLU*, the gene that encodes NAG. Currently available techniques for measuring NAG activity in patient-derived cell lines include chromogenic and fluorogenic assays and provide a biochemical method for the diagnosis of MPS IIIB. However, standard protocols require large amounts of cells, cell disruption by sonication or freeze-thawing, and normalization to the cellular protein content, resulting in an error-prone procedure that is material- and time-consuming and that produces highly variable results. Here we report a new procedure for measuring NAG activity in cultured cells. This procedure is based on the use of the fluorogenic NAG substrate, 4-Methylumbelliferyl-2-acetamido-2-deoxy-alpha-D-glucopyranoside (MUG), in a one-step cell assay that does not require cell disruption or post-assay normalization and that employs a low number of cells in 96-well plate format. We show that the NAG one-step cell assay greatly discriminates between wild-type and MPS IIIB patient-derived fibroblasts, thus providing a rapid method for the detection of deficiencies in NAG activity. We also show that the assay is sensitive to changes in NAG activity due to increases in *NAGLU* expression achieved by either overexpressing the transcription factor EB (TFEB), a master regulator of lysosomal function, or by inducing TFEB activation chemically. Because of its small format, rapidity, sensitivity and reproducibility, the NAG one-step cell assay is suitable for multiple procedures, including the high-throughput screening of chemical libraries to identify modulators of NAG expression, folding and activity, and the investigation of candidate molecules and constructs for applications in enzyme replacement therapy, gene therapy, and combination therapies.

## Introduction

Mucopolysaccharidosis type IIIB (MPS IIIB or Sanfilippo syndrome B, OMIM #252920) is an autosomal recessive lysosomal storage disorder (LSD) caused by mutations in the gene encoding the lysosomal hydrolase, N-alpha-acetylglucosaminidase (NAGLU or NAG; E.C. 3.2.1.50). NAG deficiency leads to progressive intralysosomal accumulation of the glycosaminoglycan (GAG) heparan sulfate, which, in turn, triggers a cascade of pathological events that are not yet fully understood [1]–[4]. Patients typically present with severe signs of neurodegeneration including behavioral changes and mental deterioration, which eventually leads to severe dementia and early death. To date there is no established therapeutic scheme for MPS IIIB and current treatments are largely supportive [1].

Several therapeutic approaches are being tested in cell and animal models of MPS, and a few are being translated into clinical trials or clinical practice [5]. Enzyme replacement therapy (ERT) consists of regular intravenous infusions of a recombinant enzyme that replaces the deficient enzyme and typically targets visceral organs [6]–[8]. Intrathecal injections or the use of modified recombinant enzymes able to cross the blood-brain barrier (BBB) are needed to address the neurological symptoms of MPS [9]–[11]. Substrate reduction therapy (SRT) aims at reducing the synthesis of the specific substrate that accumulates in the patient’s cells due to the catabolic enzyme deficiency [12]. Because it is based on the use of small molecules that can potentially cross the BBB, SRT represents a promising strategy to address CNS symptoms in neuropathic forms of LSDs [13]. Stop-codon read-through (SCRT) takes advantage of drugs such as aminoglycosides that are able to attenuate the termination of translation at the level of a premature STOP codon in the case of non-sense mutations. SCRT is an attractive strategy because premature STOP codons typically lack an appropriate context for an efficient termination of translation in the surrounding sequences, which enhances the selective effects of SCRT drugs leading to little consequences on normal translation while helping complete translation of the mutated protein [14]–[16]. Gene therapy (GT) is also an attractive option for MPS because it exploits the principle of cross-corrections–enzymes produced by the transduced cells are secreted and taken up by surrounding cells, including non-transduced cells, via the M6PR pathway, thus correcting cellular storage [17]–[19]. Lysosomal enhancement has been recently proposed as a general means to treat storage disorders following the discovery of a master regulator of lysosomal biogenesis and function, the transcription factor EB (TFEB) [20]–[22]. By promoting lysosomal pathways, TFEB can enhance the clearance of pathogenic storage material and thus counteract disease progression, a principle that is being demonstrated in multiple models of neurodegenerative diseases including LSDs, Huntington disease, Alzheimer disease and Parkinson disease [20], [23]–[27].

In most MPS IIIB patients, causative genetic variations within *NAGLU* are homozygous or heterozygous missense point mutations [28]–[31]. Generally speaking, missense mutations are the causative variations most frequently found in LSD patients with deficiencies in lysosomal hydrolytic activities [32], [33]. Most missense mutations do not directly impair the enzymatic function but destabilize the protein’s native structure [34]. As a result, mutated enzymes are recognized by the ER quality control system and rapidly degraded by the ER-associated degradation (ERAD) pathway [35]. The extent of degradation of enzyme variants containing misfolding, non-inactivating mutations depends on the destabilizing effect of the specific substitution [36], [37] and, in turn, determines the residual enzymatic activity in the lysosome.

Interestingly, a number of mutated enzymes retain catalytic activity if forced to fold into their native structure [38], [39]. Significant effort has been recently devoted to the development of strategies to rescue native folding of unstable mutated enzymes to prevent degradation and enhance residual enzyme activity in the lysosome. For instance, pharmacological chaperone therapy (PCT) is based on the use of small molecules that bind to the enzyme’s active site and favor native folding [37], [40]. PCT can increase the intracellular pool of active enzyme that escapes ERAD and reaches the lysosome, where the pharmacological chaperone is displaced from the enzyme’s active site due to the high concentration of substrate. As a results, PCT can effectively restore metabolic functions that are otherwise deficient in LSDs [41].

PCT candidates for LSDs have been identified by performing high-throughput screening of chemical libraries [42]–[46]. High-throughput assay capability depends on the availability of a robust and reliable assay that can be conducted in a miniaturized and automated format. However, currently available assays for measuring NAG activity *in vitro* are not suitable for high-throughput screens, since they involve large amounts of cells and several consequential steps of sample preparation. Chromogenic and fluorogenic *in vitro* assays have been developed to measure NAG activity in patient-derived fibroblasts and provide a biochemical method for the diagnosis of MPS IIIB. In the chromogenic assay, homogenates of fibroblast pellets obtained after two weeks of subculture are incubated with the colorimetric substrate p-nitrophenyl-a-D-N-acetylglucosaminide. At pH 10, the product of hydrolysis, nitrophenyl, changes color and can be quantified spectrophotometrically at 420 nm. The intensity of absorbance, normalized to the total protein content, correlates with the concentration of metabolized substrate and is thus an indication of enzymatic activity [47]. The fluorogenic assays is based on the use of 4-Methylumbelliferyl-2-acetamido-2-deoxy-alpha-D-glucopyranoside (MUG), a substrate that releases fluorescent 4-methylumbelliferone upon NAG-mediated cleavage of glycoside 1 and that has advantages in sensitivity and ease of use over the colorimetric substrate [48]. However, similar to the chromogenic assay, this method requires large amounts of cells, cell disruption by sonication or freeze-thawing, and normalization to the cellular protein content [48], [49]. Alternative and more laborious assays to measure enzyme activities in cultured fibroblasts from MPS III patients include affinity capture−release purification of biotin-tagged products followed by electrospray mass spectrometry [50]–[52] and the use of radiolabelled oligosaccharides as substrate followed by the measure of the released radioactivity to quantify NAG activity [53].

In an attempt to develop an assay amenable to high-throughput applications we developed and validated a rapid and sensitive method to quantify lysosomal NAG activity based on the use of MUG. All steps–from cell treatment with a candidate therapeutic agent to assessment of NAG activity–can be carried out in a 96-well plate, making this assay suitable for screening applications, including the identification and/or characterization of candidate therapeutics for PCT, SCRT, ERT, GT and lysosomal enhancement.

## Methods

### Cell Lines

Primary fibroblasts derived from MPS III patients were either purchased from the Coriell Institute for Medical Research (Catalog Nos. GM00156, GM00737, GM01426, GM02552 A, GM02931) or obtained from the Telethon Genetic Biobank Network, Italy (Catalog Nos. FFF0051996, FFF0071993, FFF0242004, FFF0402004, FFF0502006, FFF0631986, FFF0641986, FFF0821991). Control fibroblast cell lines from healthy donors were purchased from the Coriell Institute for Medical Research (Catalog Nos. GM03440, GM03651, GM00498).

### Reagents

All chemicals were of American Chemical Society reagent grade and, unless otherwise stated, from Sigma Aldrich (St. Louis, MO, USA). Methylumbelliferyl-2-acetamido-2-deoxy-alpha-D-glucopyranoside (MUG) was purchased from Moscerdam Substrates (Oegstgeest, Netherlands). Medium for cell culture consisted of DMEM High Glucose (HyClone), supplemented with 20% fetal bovine serum (HyClone), 2 mM L-Glutamin (Sigma) and 100 U/ml penicillin/100 µg/ml streptomycin (Sigma).

### N-acetylglucosaminidase (NAG) Activity Assay

Sample fibroblasts in culture plates were trypsinized, counted with a Neubauer hemocytometer and diluted in their standard medium to the required cell concentration. Then, 100 µl of this suspension were plated in each well of a 96-well plate (5×10^3^ or 10^4^ fibroblasts per well) and incubated at 37°C and 5% CO_2_ overnight to achieve cell attachment. Clear bottom plates (Corning, Inc.) were used. Four wells (three test wells and one background well) were plated for each condition to be tested. In order to prevent edge effects, columns 1 and 12 were filled with 100 µl PBS. Rows A and H served as background noise control to determine the extent of unspecific fluorescence and were used to incubate cells with 50 µl buffer without substrate. The medium was replaced the next day with fresh medium (containing 25 mM to 125 mM sucrose in some experiments, as indicated below) and plates were incubated at 37°C and 5% CO_2_. After treatment the medium was removed and cells washed three times with PBS. The assay reaction was started by adding 50 µl of substrate solution to each well (2 mM MUG in 0.2 M Na-acetate buffer pH 4.5 with 0.5% Triton X-100 and 1× protease inhibitor). Plates were sealed with plastic wrap to prevent evaporation, covered in aluminum foil in order to protect them from light and incubated for 17 h at 37°C. Next, 150 µl 0.2 M glycine buffer pH 10.8 were added to each well and the released fluorescence was measured (excitation 360 nm, emission 460 nm) with a Synergy 2 plate reader (BioTek). NAG activity was expressed as a fluorescence ratio between treated and untreated fibroblasts or as the percentage of wild-type enzyme activity.

### Quantitative RT-PCR

Total RNA was extracted from cells using the miRNeasy Mini Kit (Qiagen). cDNA was synthesized from 100 ng of total RNA using the QuantiTect Reverse Transcription Kit (Qiagen). Quantitative PCR reactions were performed using cDNA, PerfeCTa SYBR Green FastMix, ROX (Quanta BioSciences) and corresponding oligos [20] with the 7300 Real Time PCR System (Applied Biosystems). Samples were heated for 10 min at 95°C and amplified in 40 cycles of 1 s at 95°C and 15 s at 63°C. Expression analysis was done using SDS v1.2 software (Applied Biosystems). Threshold cycle (CT) was extracted from the PCR amplification plot. The ΔCt value was used to describe the difference between the Ct of a target gene and the Ct of the housekeeping gene: ΔCt = Ct (target gene) − Ct (housekeeping gene). Each data point was evaluated in triplicate.

### Statistical Analysis

All data were expressed as mean ± standard deviation. Statistical analyses were conducted using unpaired Student’s t-test. Differences were considered significant when p value was less than 0.05. Z′ factors were calculated according to the following equation: Z′ = 1– [3×(SD_treated_+SD_untreated_)/(Mean_treated_ – Mean_untreated_)], where SD is the standard deviation and Mean is the average of data points [54].

## Results

### Experimental Set-up for NAG 96-well Plate Cell Activity Assay

The objective of this study was to establish a fast, robust and reliable assay for measuring NAG activity, which can accommodate analysis of multiple cell lines and/or conditions at once. Towards this objective, we selected the 96-well plate as the format of choice and tested the effects of cell density and substrate concentrations on the readout of NAG activity by quantifying the fluorescence after overnight incubation of wild-type fibroblasts with the NAG-specific fluorescent substrate Methylumbelliferyl-2-acetamido-2-deoxy-alpha-D-glucopyranoside (MUG) in an acidic buffer (see Materials and Methods for details). The activity of lysosomal enzymes in cultured fibroblasts correlates with time after subculture and hence with the extent of confluency at the time of assay [55]. At confluency, fibroblasts are in a metabolic state in which highly differentiated cellular functions, including the synthesis of lysosomal hydrolases, become more important than cell division [55]. We therefore conducted our tests under conditions of cell confluency (at least 5×10^3^ cells per well in the 96-well plate format). To investigate the effect of cell density on the readout signal we compared NAG enzymatic activity of samples obtained by seeding 5×10^3^ and 10^4^ cells per well. To investigate the effects of substrate concentration on the readout signal, we tested NAG enzymatic activity by varying MUG concentration from 1 to 2.5 mM for each cell density tested. After a 17-hour incubation period, the reaction was stopped with a glycine buffer at pH 10.8 and the released fluorescence was measured on a plate reader using an excitation wavelength of 360 nm and measuring the emission at 460 nm [30].

**Figure 1:**
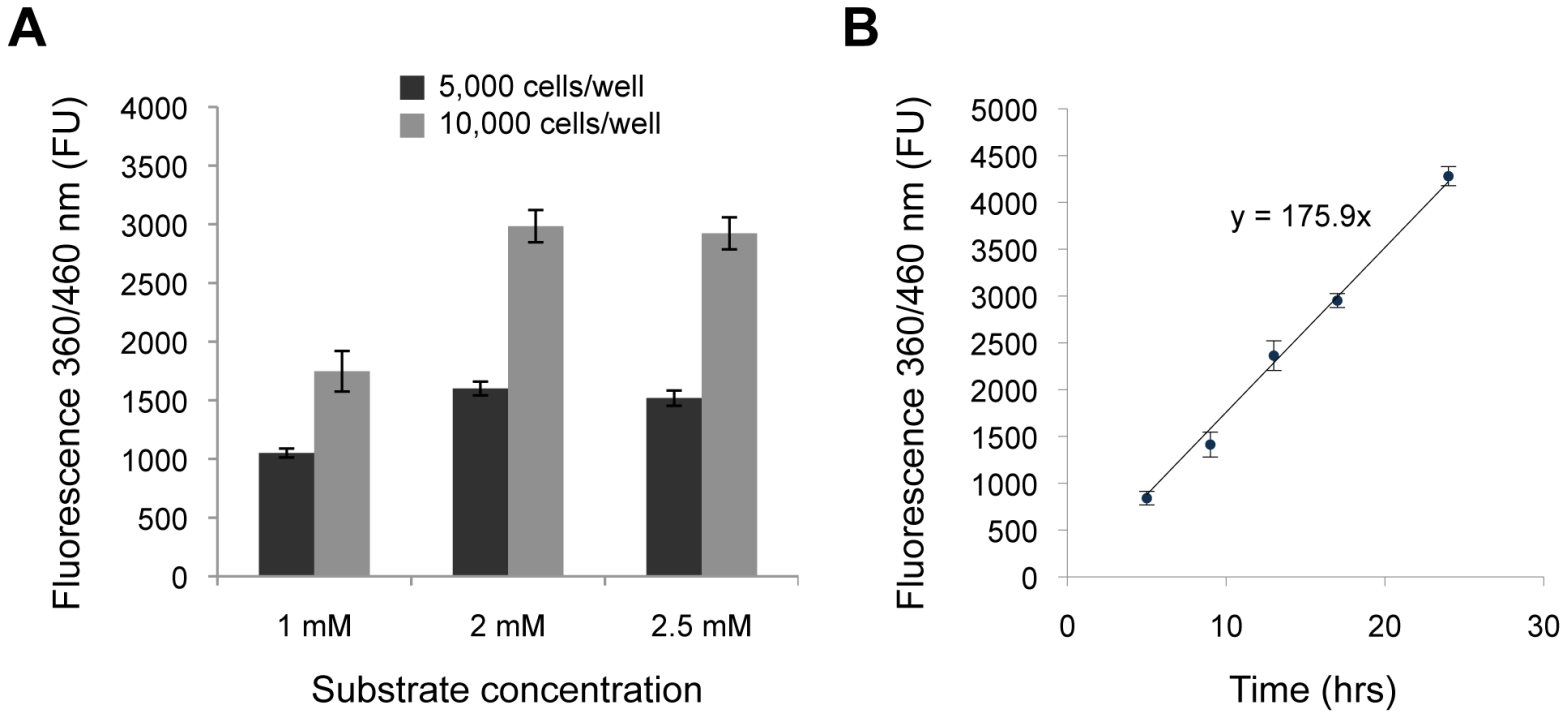
Set-up of conditions for NAG activity cell assay. **A.** Either 5 or 10×10^3^ cells were incubated for 17 hrs with different concentrations of the NAG-specific substrate, 4MU-alpha-N-acetyl-D-glucosaminide (MUG), and NAG relative activities were measured by reading the fluorescence emissions at 460 nm upon excitation at 360 nm. **B.** 10^4^ cells were incubated with 2 mM MUG and NAG relative activities were measured at various time points. FU, Fluorescence Units. Data are reported as the mean ± SD (n = 3).

Incubating 5×10^3^ fibroblasts with MUG at a final concentration of 1, 2 and 2.5 mM resulted in 1051±39, 1600±59 and 1518±65 absolute fluorescence intensity (FU), respectively. Incubating 10^4^ fibroblasts per well with MUG at a final concentration of 1, 2 and 2.5 mM resulted in 1747±172 FU, 2984±137 FU and 2923±136 FU, respectively (Fig. 1A). The measured fluorescence was higher in cells incubated with 2 mM substrate compared to cells incubated with 1 mM substrate, but did not increase further when incubated with 2.5 mM substrate, suggesting saturation of the enzymatic reaction. Hence, the combination of 10^4^ fibroblasts per well and MUG concentration of 2 mM was selected as the standard condition for subsequent experiments.

To assess whether the assay was conducted within a linear range with respect to time, we incubated 10^4^ fibroblasts with 2 mM MUG for 5, 9, 13, 17 or 24 hrs and measured NAG activity as described above. The results showed that the measured fluorescence increased linearly with time along the entire time interval tested, with an average hourly increment of 176±13 FU (Pearson correlation coefficient = 0.998; *P*<0.001) (Fig. 1B). These data indicate that the assay signal is far from reaching a plateau at the selected incubation time of 17 hrs and also suggest that shorter incubation times could be used if time is critical in the set up of the experiment.

### The NAG 96-well Plate Cell Assay Discriminates between Wild-type and MPS IIIB Fibroblasts

To assess the ability of the one-step 96-well plate cell assay to distinguish between wild-type cells and cells containing mutated enzyme variants that result in deficiencies in enzymatic activity, we measured NAG activities in fibroblast derived from 13 MPS IIIB patients and three control donors. Most MPS IIIB fibroblast lines carried *NAGLU* alleles with no residual NAG activity (L35F, V77G, Y92H, Y140C, E153K, W156C, E336X, P358L, H414R, V501G, R626X, W649C, L682R), as previously demonstrated upon transfection of plasmids with the mutated *NAGLU* cDNAs in COS-7 or CHO cells [28]–[30], [56], [57]. Another allele carried an early truncating mutation (R297X) that is also expected to result in a complete loss of NAG activity [58]. Finally, there is no published evidence reporting measurements of residual activity of three of the alleles tested (T81A, G292R, R643H).

**Figure 2:**
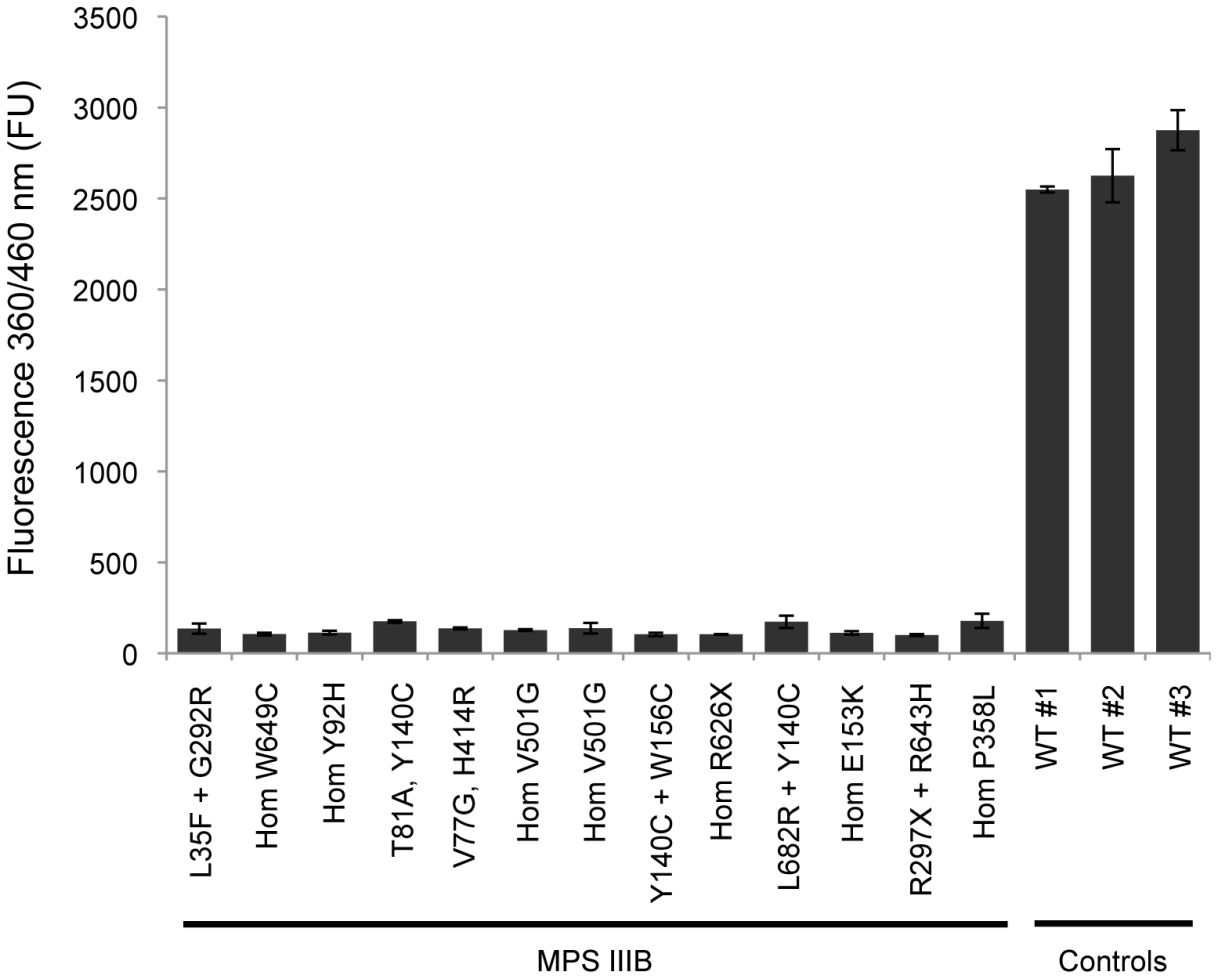
The NAG one-step cell assay discriminates between wild-type and MPS IIIB fibroblasts. Analysis of thirteen patient-derived fibroblast lines and three wild-type control lines with the NAG 96-well plate cell assay. Homozygous and compound heterozygous mutations indentified in *NAGLU* genomic sequences are indicated. FU, Fluorescence Units. Data are reported as the mean ± SD (n = 3).

The NAG cell activity assay resulted in average readouts of 2468±342 FU for wild-type fibroblasts and 131±28 FU for MPS IIIB fibroblasts, which corresponds to a ∼20-fold difference between the two groups (*P*<0.002) (Fig. 2). Differences among MPS IIIB lines or wild-type lines were not statistically significant, demonstrating that the assay is highly reliable. As previously mentioned, most of the MPS IIIB cell lines we tested carried homozygous or compound heterozygous mutations that result in no residual NAG activity. This suggests that the low signal resulting from the analysis of these cells (corresponding to 4–7% of the signal obtained from wild-type cells) represents the background noise of the assay, which is comparable to, or lower than, the level of noise from previously reported assays [28]–[30], [47]–[49], [52], [53], [56], [57], [59]. Based on the results obtained with the NAG cell activity assay, the non-characterized mutations (T81A, G292R, and R643H) are likely to confer null or very low NAG activity to the mutated NAG variants. In summary, we can conclude that the 96-well plate cell assay presents a low inherent noise content and is suitable for measuring differences in NAG activity of wild-type and MPS IIIB fibroblasts.

### Assessment of Sensitivity: Lysosomal Enhancement by Sucrose Treatment

**Figure 3:**
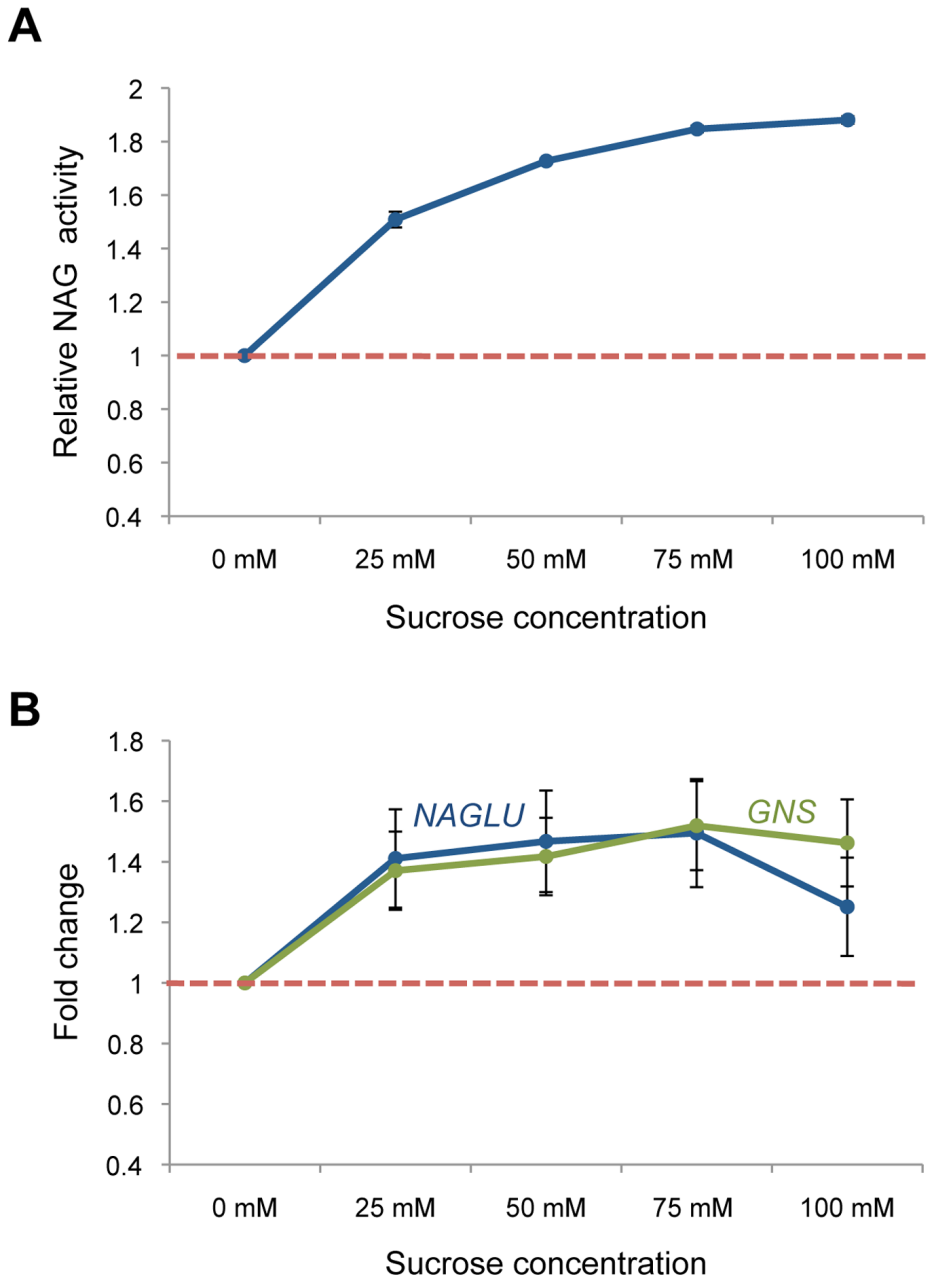
The NAG one-step cell assay detects NAG activity changes upon sucrose-mediated lysosomal enhancement. **A.** Relative NAG activity in HeLa cells treated with various concentrations of sucrose as measured with the NAG cell assay. **B.** Relative expression levels of *NAGLU* and *GNS* genes in HeLa cells treated with various concentrations of sucrose. *NAGLU* and *GNS* mRNA expression levels were obtained by real-time qRT-PCR, corrected by the expression of the housekeeping gene *GAPDH*, and normalized to those of untreated cells (red dotted line). All data are reported as the mean ± SD (n = 3).

An enzymatic assay amenable to high-throughput screening applications should be characterized by low limit of detection to reliably distinguish small differences in enzymatic activity between different variants. To assess the sensitivity of the NAG one-step cell assay, we stimulated *NAGLU* transcription via activation of the transcription factor EB (TFEB), a master regulator of lysosomal pathways [20] that directly targets *NAGLU* promoter to enhance its expression [60]. We incubated wild-type fibroblasts with sucrose, a known activator of TFEB [20], for four days at a final medium concentration of 25 to 100 mM, and measured NAG activities with the one-step cell assay as described above. The results showed a dose-dependent increase in NAG activity that reached a plateau corresponding to a 1.85 fold increase in activity at 75 mM sucrose compared to untreated cells (Fig. 3A). We also monitored the mRNA levels of *NAGLU* and of an additional lysosomal target of TFEB, namely *GNS* [20], [60], by real-time qPCR after cell treatment with sucrose under the same conditions. We observed a parallel increase in *NAGLU* expression level that reached 1.5-fold up to 75 mM sucrose compared to untreated cells (Fig. 3B). The expression of the control gene, *GNS*, also increased in a dose-dependent manner with the addition of sucrose, indicating that sucrose promoted TFEB-mediated lysosomal enhancement in these cells [20].

### Assessment of Sensitivity: Lysosomal Enhancement by TFEB Overexpression

**Figure 4:**
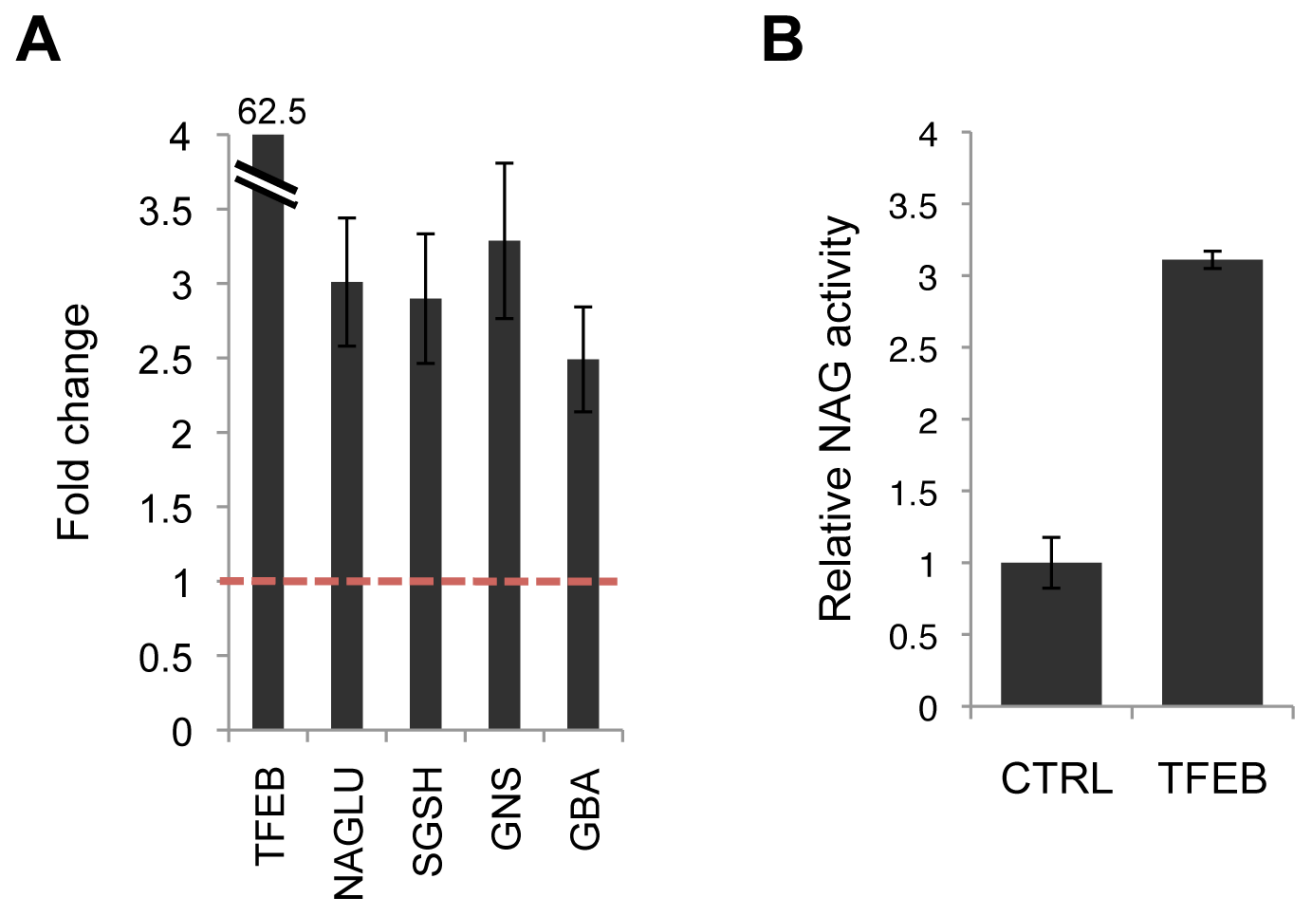
The NAG one-step cell assay detects NAG activity changes upon TFEB-mediated lysosomal enhancement. **A.** Relative expression levels of *TFEB* and four TFEB direct targets (*NAGLU*, *SGSH*, *GNS*, *GBA*) in HeLa cells upon transfection of a TFEB plasmid. *TFEB*, *NAGLU*, *SGSH*, *GNS* and *GBA* expression levels were obtained by real-time qRT-PCR, corrected by the expression of the housekeeping gene *GAPDH*, and normalized to those of untreated cells (red dotted line). **B.** Relative NAG activity in HeLa cells upon transfection of a TFEB plasmid as measured with the NAG cell assay. All data are reported as the mean ± SD (n = 3).

To determine the sensitivity of the one-step cell assay in the context of a greatly enhanced lysosomal system, we used HeLa cells stably overexpressing TFEB, in which the lysosomal compartment is significantly expanded [20]. The expression level of *TFEB* was quantified by real-time qPCR and was found to be 62.5-fold higher than that of HeLa cells not overexpressing any transgene (control cells). To confirm that the *TFEB* transgene induced the expression of its target genes in the test conditions, we quantified the mRNA levels of *NAGLU* and of other TFEB targets, namely *SGSH, GNS* and *GBA* [20], [60]. The expression of these four lysosomal enzymes was on average 2.9-fold higher in HeLa cells overexpressing TFEB than in control cells, with *NAGLU* presenting an expression 3.0 times higher than in control cells (Fig. 4A). We measured NAG activities with the one-step cell assay and found that TFEB cells displayed an activity 3.1-fold higher than control cells (Fig. 4B), thus demonstrating a close correlation between the expression of the *NAGLU* gene and NAG enzymatic activity as measured by the cell assay.

### Assessment of Reproducibility

**Figure 5:**
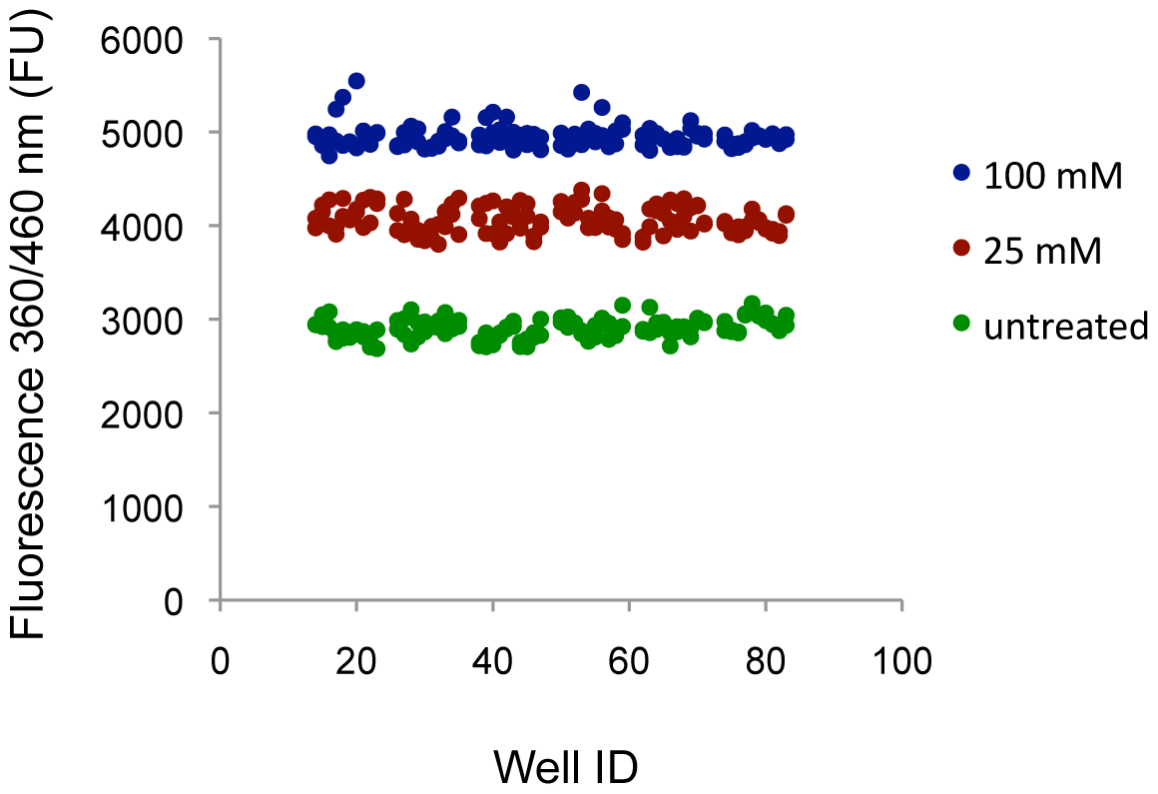
Z′-factor tests to evaluate assay reproducibility. Relative NAG activity in wild-type fibroblasts treated with 100 mM sucrose (blue dots), 25 mM sucrose (red dots), or left untreated (great dots). The graph reports the results from two independent experiments (n = 3 plates per experiment). FU, Fluorescence Units.

To verify the signal reproducibility of the NAG one-step cell assay we performed a Z′-score test in two independent experiments using three 96-well plates per experiment. Intra-plate replicates were organized following a simple scheme that included untreated cells (U) and cells treated with either 100 mM sucrose (high sucrose concentration, H) or 25 mM sucrose (low sucrose concentration, L). Columns were organized in [H-L-U], [L-U-H], and [U-H-L] schemes in plates 1, 2, and 3, respectively, to avoid any biases in the distribution of treatments. The Z′ factors generated in the two experiments were 0.73 and 0.63 in the comparison of H vs. L signals (Fig. 5), indicating that the assay presents high reproducibility of the signal and meets the requirements for high-throughput screening applications (i.e., Z′ ≥0.5) [54]. The average fold change associated with the H vs. U comparison was 1.7±0.04. The comparison of L vs. U signals generated Z′ factors equal to 0.37 and 0.38 in the two experiments, respectively, with an average fold change of 1.4±0.05. Together, these data indicate that an increase in activity equal or greater than 1.7 would reliably define a hit in the context of a high-throughput screen, whereas an increase equal or lower than 1.4 could be associated with either a hit or a false positive signal. We can take advantage of these data to provide an estimation of the minimum increase in activity required to generate a reliable hit. Our results are associated with a standard deviation of ∼5% for both untreated and treated samples. Based on the definition of Z′ score (see Material and Methods for details), we concluded that a 1.6-fold increase in NAG activity is necessary to reliably define hit compounds in the context of a high-throughput screen using the assay conditions reported in this study. Notably, TFEB overexpression resulted in a 3-fold increase in NAG activity–thus much above the threshold required to define a reliable hit, which would make TFEB a strong candidate in a genetic screen.

Based on the results obtained, we can conclude that the NAG 96-well plate assay has desirable characteristics of sensitivity and reproducibility that make it suitable for high-throughput screening applications.

## Discussion

In this study, we present a rapid, reliable and robust assay to measure NAG activity in a 96-well plate format that complements existing methods and presents desirable characteristics that make it particularly attractive for primary screening in high-throughput applications. The NAG assay herein described, in fact: (i) involves a reduced number of steps, resulting in a shorter protocol; (ii) requires a reduced number of cells, enabling the use of the 96-well plate format, which, in turn, allows testing multiple mutations, culturing and treatment conditions simultaneously and with a higher number of replicates; (iii) is performed in a single plate from start to finish, with no requirement for transfer of samples or material across plates; (iv) benefits from reduced protein inactivation due to denaturation or degradation, thus resulting in more reproducible results. On the other hand, existing methods to measure NAG activity require several steps of sample preparation that include cell disruption by sonication or repeated cycles of freeze-thawing–steps that are time consuming and difficult to standardize because they may result in protein denaturation or incomplete cell lysis, thus impacting the measured enzyme activity. As a result, the amount of cells and time typically needed for each sample–including normalization of the readout signal by DNA or protein content–are hardly compatible with a systematic testing of multiple cell lines or conditions.

In the NAG 96-well plate assay, the same number of cells is plated in each well and the number of steps involved in the experimental procedure is kept at a minimum to minimize differences in readout values due to differential cell number or growth rate. Assays conducted using 13 fibroblast lines derived from MPS IIIB patients showed that the readouts were uniformly ∼20-fold lower than control fibroblasts from healthy donors in all cases, which supports the reliability of the assay to assess deficiencies in NAG activity. In addition, upregulating NAG synthesis in HeLa cells by inducing the activation of the master lysosomal regulator, TFEB, via sucrose treatment or by direct TFEB transfection allowed evaluating the assay sensitivity to increases in NAG expression and activity. The readouts of the assay showed that changes in NAG activities paralleled changes in *NAGLU* mRNA expression as detected by real-time qPCR. Together, these data support the notion that the assay is run in conditions that are well above the background noise of the analysis and in a range of values that is far from the saturation of the signal. An assessment of reproducibility showed that the Z′ score associated with the NAG 96-well plate assay was higher than 0.6 in experiments where sucrose-mediated increase in NAG activity averaged 1.7-fold. Subsequent calculations that took into account our observed standard deviation of ∼5% showed that an increase in NAG activity ≥1.6-fold would be sufficient to reliably define a hit compound that enhances NAG activity [54]. This suggests that the NAG 96-well plate assay can be used as a primary screen in various applications to identify or investigate potential therapeutics able to modulate *NAGLU* expression or function (GT, SCRT, ERT), to rescue native folding of unstable NAG mutants (PCT), or to enhance lysosomal function–using changes in NAG activity as a sentinel readout. Hits resulting from the primary screen can be subsequently counter-screened using more labor intensive and time consuming secondary assays that take into account the effect of parameters such as toxicity and cell growth that may result in selection of false positive hits.

LSDs are perfect targets for PCT and SCRT because rescue of up to as little as 10–20% of the corresponding wild-type activity may ameliorate or even completely eliminate clinical symptoms [37], [61]. Pioneering studies that investigated SCRT in LSDs were conducted using cultured fibroblasts from patients’ skin and demonstrated that attenuating the premature termination of translation increases the activity of mutant lysosomal enzymes [14], [62]. After similar proof-of-concept studies, PCT has been the subject of intensive clinical research and it is now being translated into clinical applications for several LSDs, including Gangliosidoses GM_1_ and GM_2_, Fabry disease, Gaucher disease, and Pompe disease [42], [63]–[68]. Recently, the use of pharmacological chaperones has also been suggested as a treatment strategy for MPS IIIB [69]. In general, the modulation of the proteostasis network is a promising pharmacological strategy to promote folding of unstable, degradation-prone enzymes containing missense mutations [26], [36], [39], [70]–[74]. Pharmacological chaperones, proteostasis modulators and small molecules that induce the read-through of premature stop codons have the potential to overcome several limitations of enzyme replacement therapies (ERT): they can be ingested orally and do not require life-long invasive infusions, thus improving the patient’s quality of life at lower costs than ERT. Moreover, they can potentially cross the blood-brain barrier and thus improve the neurological phenotypes of LSDs, which are not addressed with ERT [37]. These advantages pose an urgent need to identify small molecule-based oral treatments for MPS IIIB. Due to its rapidity, sensitivity and the small format required, the NAG 96-well plate cell assay could be the method of election for screening libraries of compounds using MPS IIIB cells that carry missense or non-sense mutations. Moreover, because the stop-codon reading-through and the rescue of folding and activity of mutant proteins can be mutation-specific [14], [37], [62], [75], a positive hit that showed rescue of NAG activity with a specific mutation may be subsequently counter-screened using MPS IIIB fibroblasts carrying different mutations by cross-comparing multiple cell lines in the same 96-well cell assay. The assay is also suitable for applications aimed at investigating recombinant enzymes and constructs for gene transfer using cells with a null background, i.e. patient-derived fibroblasts. On the other hand, this assay can be used with both wild-type cells (fibroblasts or standard laboratory cells such as HeLa) and patient-derived fibroblasts to screen or characterize candidate molecules for lysosomal enhancement therapy. Importantly, combination therapies are being frequently cited as an emerging strategy to exploit the synergistic effects of multiple types of treatments [12], [16], [37], [76]–[80]. We suggest the NAG 96-well plate cell assay to be an ideal method to investigate combination therapies *in vitro*, given that the number of samples to be analyzed grows rapidly with the number of combinations tested, and even exponentially when considering multiple mutations or dosage regimens.

In summary, this assay has desirable characteristics of easy performance, rapidity, reproducibility and sensitivity that make it suitable for various applications, including the manual screening of patient-derived cells to assess residual NAG activity for diagnostic applications, the high-throughput screening of libraries of molecules to identify modulators of NAG expression, folding and activity, and the investigation of molecules and constructs to be used in ERT, GT, and combination therapies.
